# Morita Therapy-Based Nursing Support for Socially Withdrawn Japanese Youth (Hikikomori) with Gaze Phobia: A Case Report

**DOI:** 10.3390/reports9020183

**Published:** 2026-06-11

**Authors:** Mikie Ebihara, Miwa Yoshida, Kohei Handa, Katsuharu Yano, Tomoko Omiya, Kei Nakamura

**Affiliations:** 1 Department of Nursing, The Jikei University School of Medicine, 8-3-1 Kokuryo-cho, Chofu-shi 182-8570, Japan; 2 Nursing Department, The Jikei University Western Medical Center, Tokyo 201-8601, Japan; 3 Department of Psychiatry, The Jikei University School of Medicine, Tokyo 105-8461, Japan; 4 Department of Public Health Nursing, Division of Health Science, Graduate School of Medicine, Tohoku University, Sendai 980-8575, Japan; 5 The Jikei University School of Medicine, Tokyo 105-8461, Japan

**Keywords:** Morita therapy, nursing support, hikikomori, gaze phobia, purpose-driven action, social reintegration, case report

## Abstract

**Background and Clinical Significance**: “Hikikomori”—a state of prolonged social withdrawal affecting an estimated 2% of Japan’s working-age population—is frequently associated with underlying anxiety disorders, such as gaze phobia, and contributes to the socio-economic burden known as the “8050 problem,” in which aging parents support their socially isolated adult children. While Morita therapy is effective for such conditions, nursing support has historically lacked a systematic theoretical framework. This case report presents a novel nursing model analyzing the transformation process from *toraware* (mental preoccupation) toward *mokuteki-hon-i* (purpose-driven action). It proposes the ‘side-by-side’ nursing approach as a potentially important element in supporting patient autonomy in similar clinical settings. **Case Presentation**: A man in his 20s, diagnosed with gaze phobia and experiencing long-term withdrawal following traumatic bullying, was referred to our specialized short-care program. After initial preparation through structured psychoeducation regarding Morita therapy principles (toraware, sei-no-yokubo, mokuteki-hon-i), he participated in a 14-month Morita therapy-based short-care program combining individual and group interventions. Initially, the patient exhibited severe social avoidance and was trapped in a cycle of *seishin-kogo-sayo* (psychic interaction). Nurses applied ‘Strategic Inattention to Symptoms’ (*shojo-fumon*) and provided specific role suggestions, such as serving as a secretary in group discussions, to elicit his *sei-no-yokubo* (desire for life). Through the reframing of his anxiety as a constructive drive, the patient shifted to a purpose-driven stance. Outcomes showed improved self-adjustment skills in public spaces and successful social reintegration through sustained part-time employment. **Conclusions**: Nursing care characterized by ‘intentional non-intervention’—which involves waiting in a ‘side-by-side’ manner within a minimally structured environment—may contribute to fostering patient autonomy in similar clinical contexts. This ‘experience-oriented’ approach appeared to elicit inner strengths and support self-regulation in this case, warranting further investigation in multi-case designs. The relative contributions of individual nursing support and group therapeutic milieu cannot be disentangled in a single-case design.

## 1. Introduction and Clinical Significance

Recent social data from Japan indicate that approximately 40% of individuals in their 20s and 30s experience profound loneliness, the highest rate among all generations [1]. Nationwide estimates suggest that approximately 1.46 million individuals currently live in a state of hikikomori, representing 2.05% of the population aged 15–39 and 2.02% of those aged 40–64 [2]. Such isolation may, in some cases, manifest as hikikomori, a state of social withdrawal lasting six months or longer, typically emerging by the late 20s [3]. This condition is frequently associated with underlying psychiatric disorders, including anxiety disorders and obsessive–compulsive and related disorders [4,5]. Persistent hikikomori contributes to Japan’s ‘8050 problem’ of aging parents supporting socially isolated adult children, creating significant familial burden [5]. Individualized clinical interventions are urgently needed; the present report focuses on one such approach at the individual level.

Although the term hikikomori is Japanese and carries specific cultural connotations, the phenomenon of severe, prolonged social withdrawal is not confined to Japan. Early literature introducing the concept was careful to draw this distinction. Subsequent international research has documented hikikomori-like presentations in Hong Kong, Spain, India, Oman, South Korea, the United States, and numerous other countries [6]. A 2023 meta-analysis of 19 studies covering more than 58,000 participants estimated a pooled global prevalence of approximately 8% [7]. In other words: the word hikikomori travels with cultural caveats, but the condition travels without them. The present case report is therefore offered not only to Japanese clinicians but to practitioners in any setting who work with young people experiencing severe, prolonged social withdrawal.

Morita therapy, developed in Japan in the early 1900s, is an acceptance-based psychotherapy with parallels to mindfulness-based therapies and Acceptance and Commitment Therapy (ACT) that focuses on accepting symptoms as they are (*aru-ga-mama*) [8]. Unlike therapies seeking symptom reduction, Morita therapy interprets anxiety as intrinsically linked to the *sei-no-yokubo* (desire for life)—the innate drive to live better [9,10]. Pathological states are viewed as *toraware* (mental preoccupation), in which attempts to suppress natural emotional experiences perpetuate a *seishin-kogo-sayo* (psychic interaction), a vicious cycle of heightened attention and sensation [11]. The therapeutic goal is a transition to a *mokuteki-hon-i* (purpose-driven action) stance, where individuals engage in life tasks despite subjective discomfort [9].

Historically an inpatient treatment, Morita therapy has recently shifted toward multi-disciplinary outpatient and short-care settings [12,13]. Its application has expanded to diverse fields, including internet addiction [14], alcohol use disorder [15], and terminal care [16]. In daycare and short-care settings, group-based Morita therapy approaches have been reported to support the deconstruction of toraware and to be associated with improvements in GAF scores in clinical case series and program reports [17,18], though controlled evidence in hikikomori-specific populations remains limited.

In these settings, nurses serve as companions in a ‘side-by-side’ relationship, supporting autonomy through shared work [19]. However, while multidisciplinary support is effective, the specific nursing mechanisms that stimulate the Desire for Life during the work process remain underexplored, as existing studies have primarily focused on home-visit support [20]. This study aims to analyze the nursing process for a hikikomori patient with gaze phobia, exploring how interventions based on ‘Strategic Inattention to Symptoms’ (shojo-fumon) guide the patient toward Purpose-driven Action.

This case report establishes a novel nursing model by demonstrating how a therapeutic environment that is minimally structured can foster self-adjustment skills. It highlights that a nurse’s ‘intentional non-intervention’—waiting for the patient’s initiative—may represent a professional technique relevant to restoring patient subjectivity in this clinical context. These findings offer a practical framework for clinical practitioners to support the social reintegration of hikikomori patients and alleviate the multi-layered pressures faced by their families within the context of Japan’s ‘8050 problem’.

## 2. Case Presentation

### 2.1. Theoretical Framework and Conceptual Definitions

This study is grounded in the principles of Morita therapy, which emphasizes accepting symptoms as they are (aru-ga-mama). Within this framework, several core concepts define the recovery mechanism. The Desire for Life (sei-no-yokubo) is the innate drive to live better that exists behind anxiety, serving as the primary motivation for recovery. Mental Preoccupation (toraware) is a pathological state in which attention becomes fixed on natural emotional experiences because they are regarded as abnormal and targeted for elimination. This fixation triggers Psychic Interaction (seishin-kogo-sayo), a vicious cycle in which focusing on a sensation intensifies it, thereby attracting further attention. The therapeutic goal is to establish Purpose-driven Action (mokuteki-hon-i), a stance in which the individual engages in necessary life tasks despite subjective discomfort, prioritizing objectives over current emotional states.

Note: The term ‘Strategic Inattention to Symptoms’ is used throughout this report as the English rendering of shojo-fumon. The literature also uses ‘non-attention to symptoms’ [19] and ‘letting the symptom be.’ Our rendering was chosen to emphasize the intentional, clinical nature of the stance. All three translations refer to the same therapeutic posture; see Table 1 for a full glossary.

### 2.2. Methodology and Analysis

This study employed a qualitative case study design focusing on a single patient’s recovery process to analyze the mechanism of deconstructing (*toraware*) through specific Thanknursing interactions. To ensure academic rigor and the quality of the nursing practice description, the study design and analysis were conducted in accordance with the review criteria for nursing practice case studies proposed by Yamamoto [22].

Following the task analysis framework proposed by Greenberg [23], the boundaries of the case were clarified, and both the nurse’s intervention intentions and the patient’s transformation elements were extracted. The primary data consisted of process records created using the “reconstruction of nursing situations” method [24], which involves documenting verbal and non-verbal reactions alongside the nurse’s internal decision-making processes through reflection on specific interactions.

A total of 29 process records were analyzed, covering all sessions across the 14-month intervention. Transformation elements were operationally defined as moments in which Patient A’s verbal or non-verbal behavior showed a discernible shift from symptom-focused avoidance toward goal-directed action. Nurse C acknowledges that negative or ambiguous episodes were not systematically documented in the process records; this represents a potential source of confirmation bias and is identified as a limitation. 

Regarding the three-phase structure, we acknowledge that Nurse C was familiar with prior Morita therapy nursing literature, including case reports employing phase-based recovery frameworks, before the process records were written. The three-phase structure therefore reflects a hybrid analytic approach: an a priori theoretical schema informed initial interpretation, which was subsequently refined through systematic review of the process record data. Readers should weight the analytic claims accordingly: the three-phase structure is best understood as a theoretically informed interpretive framework applied to the case, rather than as a structure that emerged purely from the data.

To ensure data integrity, process records were written by Nurse C within 24 h of each session, prior to any formal data analysis.

The authors acknowledge that Nurse C served as both the primary clinician performing the intervention and the source of primary data, representing a potential conflict of interest. To mitigate this limitation, all analytical interpretations were independently verified by T.O., an expert with a public and community mental health perspective, who had no direct involvement in the clinical intervention.

Discrepancies between Nurse C and the independent verifier were resolved through structured discussions. Specifically, in accordance with Yamamoto’s guidelines for ensuring validity in case studies [22], interpretations were refined over two group sessions involving all authors and one additional consultation until a consensus was reached. All interpretive inconsistencies were successfully resolved. This report adheres to the CARE checklist guidelines [25].

### 2.3. Case Profile and Intervention Context

#### 2.3.1. Case Selection Rationale

Patient A was selected as a representative case because he met the operational criteria for hikikomori (social withdrawal exceeding six months [3]), carried a primary psychiatric diagnosis of gaze phobia, and demonstrated all three theoretically proposed phases of the Morita therapy-based nursing process across a complete 14-month intervention.

The international literature increasingly frames prolonged social withdrawal as transdiagnostic and biopsychosocial in origin, involving interactions among temperamental factors, family dynamics, educational and labour-market structures, digital ecology, and macroeconomic precarity [5]. Bullying-precipitated social anxiety, as presented by Patient A, represents one pathway into hikikomori among several. The nursing model proposed in this report is most clearly grounded in this specific pathway. Its applicability to withdrawal arising from other origins—including neurodevelopmental conditions, mood disorders, or demoralization—is a hypothesis for future investigation, not a claim of the present paper.

This case was not selected on the basis of exceptional outcomes; rather, it was identified as clinically typical of the hikikomori patients referred to this short-care program in that he presented with comorbid social anxiety, a history of school-based trauma, and progressive functional deterioration prior to referral. We acknowledge, however, that as the first formally analyzed case within this nursing model, its representativeness relative to the broader hikikomori population cannot be determined without future multi-case comparison.

#### 2.3.2. Patient Background

Patient A was formally diagnosed with Gaze Phobia under DSM-5 Social Anxiety Disorder (SAD). While the phenomenology of his presentation shares features with the Japanese clinical concept of *taijin kyofusho*, the diagnosis was made according to DSM-5 criteria and classified as SAD.

He presented for his initial outpatient psychiatric consultation to the psychiatric outpatient clinic in Y Month 20XX. Following traumatic bullying in middle school, he began experiencing prolonged absence from school, eventually leading to complete school dropout. The total duration of pre-intervention social withdrawal was approximately five years, from the onset of school dropout following bullying to the initial psychiatric consultation. As his school attendance dropped, his interactions with peers gradually diminished, and he became increasingly isolated from his social network. This resulted in a state of long-term social withdrawal (hikikomori), where he spent most of his time confined to his room, with progressive loss of social roles and self-efficacy. He typically left his home only at night to avoid the gaze of others.

#### 2.3.3. Pre-Intervention Preparation and Pharmacotherapy

Prior to entering the short-term care program, Patient A underwent a preparation phase consisting of structured psychoeducation regarding Morita therapy principles. No pharmacotherapy was administered; the patient remained drug-free throughout the preparation phase and the 14-month intervention. Regarding other psychological interventions, although the patient had previously received Cognitive Behavioral Therapy (CBT) at a former hospital, neither CBT nor any other forms of counseling were conducted at our institution. All clinical decisions were managed by the supervising psychiatrists. The absence of concurrent pharmacotherapy or psychotherapy at this institution means that the observed outcomes are less likely to reflect pharmacological confounding; however, prior CBT at another hospital and other patient-specific factors cannot be excluded.

The psychoeducation component comprised approximately three to four individual sessions prior to program entry, in which the nurse introduced core Morita therapy concepts (*toraware*, *sei-no-yokubo*, *mokuteki-hon-i*) using standardized program materials [10]. The authors acknowledge that this pre-intervention preparation may have contributed to the patient’s initial capacity for engagement with the program.

#### 2.3.4. Intervention Setting and Group Context

The intervention was conducted in a psychiatric short-care facility affiliated with a hospital under Japanese Medical Law. Operating as a therapeutic community, the environment was managed by a multidisciplinary team—including physicians, nurses, and clinical psychologists—monitoring group dynamics while providing individualized symptom observation. The program involved 3-h sessions with approximately 15 participants ranging from their 20s to 50s, primarily diagnosed with Anxiety Disorders (e.g., Social Anxiety Disorder) or conditions historically classified as neurotic disorders, such as OCD and related disorders, with frequent comorbid Depressive Disorders. The environment was managed following the clinical standards established for modern Morita therapy [26].

No formal family meetings were convened as a component of this programme. Although Patient A’s parents received information through the multidisciplinary team, they did not directly participate in the short-term care intervention. Patient A reported experiencing gaze phobia toward family members as well as in non-familial contexts; however, the family did not seek medical consultation regarding this issue. Despite this, Patient A described generally constructive domestic interactions, including academic support from a sibling and shared recreational activities. The parents demonstrated a supportive and permissive attitude, respecting the patient’s intentions and providing logistical and financial support, including the father driving the patient to and from the university.

Participants autonomously decided on activities—such as pottery, cooking, gardening, or handicrafts—and negotiated role assignments. The therapeutic goal was to foster both a ‘Strategic Inattention to Symptoms’ (shojo-fumon) stance—in which participants redirect attention from symptoms toward purposeful activity—and a purpose-driven action (*mokuteki-hon-i*) orientation, emphasizing the completion of self-determined tasks even in the presence of anxiety. The authors acknowledge that group dynamics, including the influence of peer participants who modeled Purpose-driven Action, inevitably contributed to Patient A’s therapeutic progress; systematic analysis of group-level effects falls outside the scope of this single-case report and is identified as a limitation.

The intervening nurse, Nurse C, was a specialist with over ten years of clinical experience in Morita therapy-based care and had undergone extensive professional training. The total intervention period spanned 14 months (Year Y to Y + 14), comprising 29 sessions in total (Table 2).

### 2.4. The Morita Therapy-Based Nursing Support Process (See Figure 1)

The nursing support process was characterized by a deliberate transition from an initial stance of waiting with strategic inattention to the proactive proposal of social roles, ultimately fostering the patient’s self-regulation by maintaining an environment that was not overly structured. The nursing support for Patient A spanned 14 months Month 1 to Month 14 and was analyzed across three distinct phases: the Introductory, Transition, and Consolidation phases.

#### 2.4.1. Phase 1: Breaking the Vicious Cycle (Introductory Phase: Month 1)

During Session 1 (Month 1), Patient A exhibited severe interpersonal avoidance and performed individual tasks in isolation. He asked, “*Is it okay if I continue working alone?*”. Nurse C adopted a stance of ‘Strategic Inattention to Symptoms’ (shojo-fumon), responding with “Let’s see how it goes.” This intentional non-intervention aimed to establish a foundation of trust and disrupted the Psychic Interaction cycle (seishin-kogo-sayo) by not validating the symptom as a problem.

*“Is it okay if I continue working alone?”*

**(Patient** **A)**

*“Let’s see how it goes.”*

**(Nurse** **C)**

The nurse practised ‘Strategic Inattention to Symptoms’ (shojo-fumon), refraining from exploring the details of his anxiety.

#### 2.4.2. Phase 2: Eliciting the Desire for Life (Transition Phase: Months 3–7)

By Session 9, Patient A expressed a desire for more active participation despite his anxiety. Rather than engaging with his distress, Nurse C redirected his attention toward a constructive behaviour she had observed—his use of a smartphone to research discussion topics—and proposed that he serve as the group secretary.

This redirected his focus from internal symptoms toward external, goal-directed activity, eliciting his innate *sei-no-yokubo* (desire for life). Redefining Emotions (Session 11, Month 5): When Patient A felt guilty about joining the group discussions, the nurse redefined his guilt as a constructive desire to cooperate with others. By Session 17 (Month 7), despite arriving late due to feeling unwell on the train, he was able to participate in pottery and reported finding the interaction enjoyable.

*“I’m afraid I’m staring too much at others in discussions. I want to be more active, but I’m worried about causing discomfort.”*

**(Patient** **A)**

*“I noticed you were researching topics on your smartphone and contributing ideas during the task. Why not try serving as the secretary for the discussion?”*

**(Nurse** **C)**

Nurse C avoided symptom-centered dialogue and instead highlighted his constructive actions, reallocating his attention toward external goals and eliciting his innate Desire for Life.

“Maybe I’ll try it. I wonder if the group will think I am motivated.”

**(Patient** **A)**

#### 2.4.3. Phase 3: Fostering Self-Adjustment (Consolidation Phase: Months 11–14)

Between Sessions 17 and 18, a four-month interval elapsed during which Patient A did not attend the short-care program. During this period, due to hikikomori symptoms, he was unable to attend outpatient psychiatric appointments at our institution; no formal nursing interactions were conducted.

During Session 18 (Month 11), Patient A reported that he had independently initiated part-time employment in a customer-facing retail role. This decision was not prompted by a prior nursing suggestion; in Session 17, Nurse C had validated Patient A’s growing confidence in public spaces but made no specific recommendation regarding employment. The employment decision therefore appears to reflect autonomous generalization of the self-regulation skills developed during the intervention—consistent with the Purpose-driven Action stance—though the absence of session records during this interval limits certainty about the contributing factors. This four-month gap of unobserved recovery is acknowledged as a limitation (see Section 3.4).

In Session 21 (Month 12), Patient A demonstrated an increased capacity for autonomous decision-making and awareness.

*“When I’m playing table tennis, I don’t notice the gaze of others.”*

**(Patient** **A)**

This realization embodied the ‘experience-oriented’ nature of recovery where attention shifts naturally from self to task.

By Session 29 (Month 14), he demonstrated consolidation of therapeutic gains.

*“I felt unwell on the train, so I decided to get off and rest. That’s why I’m late today.”*

**(Patient** **A)**

He subsequently fulfilled his duties as the day-duty officer—a rotating leadership role responsible for opening the session and facilitating group coordination—upon arrival. By maintaining a minimally structured environment and refusing to provide ready-made solutions, the nurse fostered Patient A’s self-adjustment skills. This intervention solidified his Purpose-driven Action (*mokuteki-hon-i*) stance. Nurse C validated this process, noting that his actions were the result of conscious self-regulation.

*“It’s important that you judged for yourself to rest. It’s wonderful that your actions did not drop to zero.”*

**(Nurse C)**

### 2.5. Outcome: The Patient’s Recovery Process

**Figure 1:**
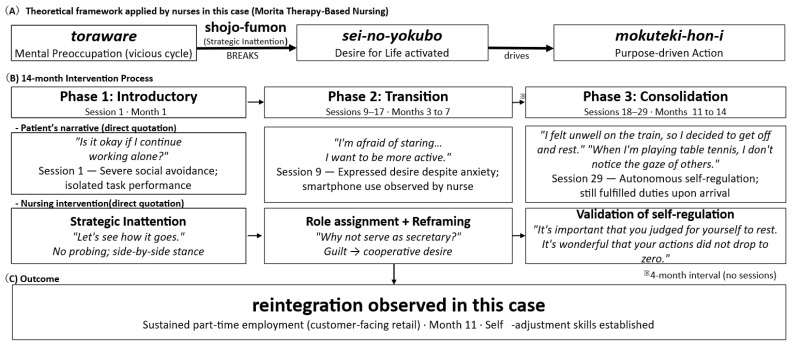
The Morita therapy-based nursing support process for Patient A across three intervention phases. (**A**) Theoretical framework applied by nurses: shojo-fumon (strategic inattention) disrupts toraware (mental preoccupation), activating sei-no-yokubo (desire for life) and driving mokuteki-hon-i (purpose-driven action). (**B**) The three-phase nursing support process across 14 months, highlighting representative patient narratives and nursing responses (note: a four-month interval occurred between Phase 2 and Phase 3). (**C**) Outcome observed: sustained part-time employment and establishment of self-adjustment skills (Month 11).

Through the 14-month intervention, from Month 1 to Month 14, Patient A successfully established a Purpose-driven Action stance. As summarized in Figure 1, this recovery process was characterized by three critical developments: (1) the disruption of the psychic interaction cycle through strategic inattention, (2) the elicitation of the Desire for Life through role activation and emotional reframing, and (3) the consolidation of self-adjustment skills through the validation of autonomous judgment, ultimately culminating in sustained social reintegration.

Regarding standardized outcome measures, validated psychometric instruments—such as the Liebowitz Social Anxiety Scale (LSAS), GAF, or the hikikomori Questionnaire (HQ-25)—were not prospectively administered during the intervention period, as formal pre/post assessment was not part of routine clinical practice at this program at the time. Clinical progress was therefore evaluated on the basis of qualitative behavioral changes and the patient’s self-reported shift toward a purpose-driven lifestyle, as documented in the process records. The absence of standardized measures precludes quantitative assessment of symptom change and is acknowledged as a limitation of the current report (see Section 3.4).

These core developments unfolded as follows:

First, the deconstruction of Psychic Interaction occurred as his attention shifted naturally from his internal state to external tasks during group activities. This change was epitomized by his realization:

*“I don’t notice my gaze when I’m playing table tennis.”*

**(Patient** **A)**

Second, his subjective distress was redefined as a hidden desire to cooperate, allowing him to accept his nervousness as a natural part of his character in a state of aru-ga-mama. His emerging Desire for Life was clearly articulated through his own words:

*“I want to be able to communicate better.”*

**(Patient** **A)**

Finally, the recovery culminated in the establishment of self-adjustment skills and successful social reintegration. By Month 11, he had prioritized his long-term goal of social participation over his short-term moods, leading to sustained part-time employment. The patient’s recovery process manifested as a structural shift from pathological fixation to a dynamic, action-oriented lifestyle, facilitated by the deconstruction of toraware and the activation of sei-no-yokubo.

The primary functional outcome was Patient A’s sustained part-time employment in a customer-facing retail role (approximately 3–4 h per shift, 2–3 days per week), which he initiated autonomously at Month 11 and maintained through the conclusion of the intervention at Month 14. Program attendance across the 14-month period was full (29 of 29 scheduled sessions); although the patient arrived late to Session 17 due to physical discomfort on the train, he participated in the session’s activities.

No adverse events were observed or reported during the intervention period. By Phase 3, Patient A reported being able to use public transportation independently, including during peak hours; no participation in other social activities beyond part-time employment was reported during the intervention period. Family involvement was limited to information sharing via the multidisciplinary team. While the authors treat Patient A’s sustained employment as a functionally meaningful outcome, this should not be interpreted as evidence of comprehensive social reintegration, as systematic assessment of social functioning across multiple domains was not conducted.

In accordance with ethical best practices for case report publication [25], Patient A reviewed the final manuscript prior to submission in February 2026 for factual accuracy and identifiability. He was not formally invited to comment on the interpretive framing of his recovery process; whether the authors’ account of his transformation reflects his own self-understanding is therefore unknown. This is identified as a limitation in Section 3.4.

## 3. Discussion

The findings of this case report suggest that nursing intervention based on Morita therapy may be effective in facilitating the social reintegration of individuals with hikikomori and social anxiety.

### 3.1. Redefining “Waiting” as Intentional Non-Intervention

The nursing practice in this study, conducted within a short-care setting, indicates that waiting constitutes not merely a passive stance but a specialized technique of ‘intentional non-intervention’. By deliberately securing space for the patient to autonomously face and navigate anxiety, Nurse C’s response to Patient A—“ Let’s see how it goes (Session 1)”—functioned as a catalyst for the patient’s self-adjustment skills. This aligns with previous studies [19,20] suggesting that when supporters refrain from over-directing and instead patiently wait for the patient’s initiative, it becomes a crucial turning point for social recovery. Furthermore, this stance provides a safe therapeutic space where patients feel psychologically safe to fail. In this environment, Patient A was permitted to remain symptomatic or perform tasks imperfectly without fear of judgment. This safety is the active acceptance of the patient’s current state, allowing them to engage in trial and error as a prerequisite for self-adjustment skills.

### 3.2. Purpose-Driven Stance as a Bridge Between Clinical and Social Life

The interventions in short-term care serve as a preparatory stage (Step 2–3) [12,13] within a multimodal intervention framework for social reintegration. Patient A’s ability to autonomously decide to get off the train when feeling unwell (Session 29) exemplifies successful adaptation. By de-emphasizing the outcome of arriving late and instead valuing the process of the patient’s decision-making, the nurse reinforced a practical self-care strategy. This suggests that the waiting approach in a clinical setting may support autonomous self-care behavior in daily life.

### 3.3. ‘Side-by-Side’ Accompaniment in an Urban Digital Society

The ‘side-by-side’ approach found in this case is a nursing stance that eliminates vertical hierarchies and accompanies the patient within the same experiential horizon of daily challenges. This maintains an optimal distance that restores autonomy without imposing excessive pressure. Notably, Patient A’s use of a smartphone to express opinions (Session 9) suggests that digital tools can serve as a low-threshold gateway for those with interpersonal anxiety. Re-framing this action as a *sei-no-yokubo* (desire for life) and assigning a specific role (e.g., secretary) shifted the patient’s attention from internal anxiety to external tasks, effectively eliciting his latent altruistic desire to be helpful to others. This shift from self-preoccupation to social contribution is a distinctive therapeutic mechanism of Morita therapy that bridges the gap between clinical recovery and social reintegration.

Of the nursing features described in this case, some are likely to be transdiagnostic: the side-by-side stance, strategic inattention to symptoms, and the gentle redirection of attention from self-preoccupation toward purposeful external tasks may speak to withdrawal arising from diverse origins. Others appear more pathway-specific: the reframing of gaze-related guilt as a latent desire for cooperativeness relies on a cognitive schema more characteristic of SAD-spectrum presentations. This distinction should guide future clinical adaptation of this model.

This ‘experience-oriented’ approach is a promising model for promoting social reintegration while bolstering self-esteem in an increasingly digitalized and socially isolated society. Furthermore, it bridges Morita therapy with global psychological frameworks.

The therapeutic moves described in this case are not parochial to Morita therapy or to Japanese clinical practice. The move Nurse C made in Session 9—declining to engage with the patient’s gaze-related distress and instead proposing the role of secretary—is structurally analogous to Frankl’s technique of dereflection: redirecting attention away from the symptom toward an externally meaningful task. It also resonates with ACT’s pairing of defusion (disengaging from the literal content of anxious thoughts) with committed action (moving toward valued behavior despite discomfort) [27]. Behavioral activation similarly targets the same shift: from inward rumination to outward engagement. However, Morita therapy, ACT, and mindfulness-based therapies should be understood as convergent rather than equivalent: each reaches similar clinical moves through distinct theoretical routes, and important differences in mechanism and practice remain. Morita therapy does not employ formal defusion techniques or values clarification exercises, and aru-ga-mama, while sharing a surface resemblance with mindfulness acceptance, does not involve formal meditation practice. Morita therapy uniquely reframes client resistance as a latent desire for recovery [28]. Furthermore, as Kirmayer emphasizes, culturally grounded interventions are essential for understanding the person within their specific social context, particularly in non-Western societies [29].

Given the single-case qualitative design, all interpretations presented in this report should be understood as hypothesis-generating. The observed outcomes reflect the combined effects of Morita psychoeducation, the therapeutic milieu, peer modeling, repeated exposure to social situations, patient motivation, and the nursing interventions described. Replication across multiple cases with standardized assessment is necessary before conclusions about causal mechanisms or clinical generalizability can be drawn.

### 3.4. Limitations and Future Research

First, as a qualitative single-case analysis, the findings are exploratory and hypothesis-generating; they do not permit causal inference or generalization to broader hikikomori populations. Case selection was based on theoretical purposiveness rather than random sampling, and the influence of patient-specific factors—including pre-existing motivation, the effects of pre-intervention psychoeducation, and individual pharmacological response—cannot be disaggregated from the effects of the nursing intervention.

Second, while no concurrent pharmacotherapy was administered throughout the 14-month intervention, this means the observed outcomes reflect a drug-free recovery process. Although this eliminates pharmacological confounding variables, the findings may not be directly applicable to patients requiring medication for symptom management. Furthermore, while the patient had previously received Cognitive Behavioral Therapy (CBT) at another hospital, no such interventions were conducted during this study, focusing the results solely on Morita therapy-based care.

Third, the primary data source consisted of process records created retrospectively—though within 24 h of each session—by the intervening nurse, who also served as the primary data analyst. Despite independent expert verification, this dual role introduces the possibility of confirmation bias and limits the objectivity of the data.

Specifically, negative or ambiguous clinical episodes were not systematically documented, and the patient’s own interpretive perspective on the recovery process was not formally collected, both of which would have strengthened the analytic rigor of this report.

Fourth, standardized psychometric assessments (e.g., LSAS, GAF) were not prospectively administered during the intervention period, precluding quantitative measurement. Future replications should incorporate at minimum one validated instrument at baseline, intervention end, and follow-up; the HQ-25, purpose-built for hikikomori populations, would substantially strengthen both within-case documentation and cross-case comparability.

Fifth, the group dynamics were not systematically analyzed and may have substantially contributed to Patient A’s progress. The process records focused on dyadic nurse-patient interactions and did not capture peer-level exchanges. No method was available to disentangle the effect of individual nursing support from group-level peer effects. The observed improvement should therefore be understood as reflecting the combined therapeutic milieu, rather than the isolated effect of any single nursing intervention.

Sixth, a four-month interval between Sessions 17 and 18 represents a period of unobserved recovery. As Patient A was unable to attend outpatient appointments due to hikikomori symptoms during this interval, no formal nursing process records were created, and the factors contributing to his autonomous decision to initiate employment cannot be fully determined.

Seventh, Patient A reviewed the final manuscript for factual accuracy and identifiability but was not formally invited to comment on the interpretive framing of his recovery process. Future case reports in this tradition should consider incorporating a formal patient reflective interview as part of the analytic process.

Finally, the clinical application of ‘intentional non-intervention’ may not be appropriate for all patients or symptom profiles. Patients with severe anxiety manifesting as physical fears (e.g., heatstroke during commutes) or compulsive symptomatic behaviors (e.g., repetitive handwashing) may be overwhelmed rather than supported by a non-directive nursing stance, suggesting that this approach must be carefully calibrated to symptom severity and the patient’s current capacity for autonomous functioning.

Future research should involve prospective multi-case designs with standardized assessments at baseline and follow-up, formal documentation protocols for concurrent pharmacotherapy, and investigation of the moderating effects of urban digital communication tools as low-threshold entry points for social interaction. Long-term evaluations of nursing’s role within multimodal interventions involving both patients and their families are also needed.

## 4. Conclusions

This case study suggests that nursing interventions rooted in Morita therapy—specifically ‘intentional non-intervention’ and ‘side-by-side’ accompaniment—may be valuable for promoting the social reintegration of patients with hikikomori, though this interpretation is necessarily hypothesis-generating given the single-case qualitative design.

The central clinical implication suggested by this case is that nurses might consider not focusing on symptom reduction, but rather on fostering the patient’s desire for life/*sei-no-yokubo* through shared activities and deliberate, patient-centered waiting. By valuing the patient’s autonomous decision-making process over clinical outcomes (such as punctuality), nurses can bridge the gap between clinical support and independent living in an increasingly digitalized and socially isolated society.

For practitioners outside Japan who may wish to adapt this approach, the shojo-fumon posture is, in our experience, teachable through a combination of theoretical study of Morita therapy principles, structured clinical supervision, and reflective practice using process records. Immersion within a Japanese therapeutic community, while enriching, does not appear to be a prerequisite. The Japanese Society for Morita Therapy’s outpatient guidelines [10] provide a systematic clinical reference. We invite colleagues internationally to engage with this framework, to test its adaptation across cultural and diagnostic contexts, and to report their findings.

## Figures and Tables

**Table 1:** Glossary of Core Morita Therapy Terms Used in This Report.

Japanese Term	Literal Translation	Clinical Paraphrase	Closest Western Analogue
*toraware*	Being caught/mental preoccupation	Pathological fixation on a symptom, intensified by the very act of attending to it	Cognitive fusion (ACT); attentional bias (CBT)
*sei-no-yokubo*	Desire for life	The innate drive to live better that lies behind anxious symptoms; the primary motivation for recovery	Will to meaning (Frankl); values-as-direction (ACT)
*mokuteki-hon-i*	Purpose-first/purpose-driven action	Engaging in valued life tasks despite subjective discomfort, prioritizing objectives over current emotional state	Committed action (ACT); behavioral activation (CBT)
*aru-ga-mama*	As it is	Accepting one’s current state—including anxiety—without forcing change or suppression	Radical acceptance (DBT); present-moment acceptance (ACT/mindfulness)—note: not equivalent to formal meditation practice
*shojo-fumon*	Non-attention to the symptom/strategic inattention	Clinician deliberately refrains from engaging symptom content; redirects attention toward purposeful activity	Dereflection (Frankl); defusion (ACT)
*seishin-kogo-sayo*	Psychic interaction	Vicious cycle in which focusing on a sensation intensifies it, which in turn attracts further attention	Attentional amplification; symptom maintenance cycle (CBT)

Glossary of Core Morita Therapy Terms Used in This Report [8,9,10,21]. Definitions and clinical paraphrases are primarily based on Morita [8], Kitanishi [9], Japanese Society for Morita Therapy [10], and Nakamura et al. [21]. Western analogues are identified for orientation only; these frameworks should be understood as convergent rather than equivalent (see Section 3.3). The term ‘Strategic Inattention to Symptoms’ as a rendering of shojo-fumon follows Asanuma [19]; see Section 2.1 footnote.

**Table 2:** Clinical timeline of the Morita therapy-based short-care intervention for Patient A.

Session No.	Period/Phase	Milestone	Key Nursing Action	Patient Behavioral Outcome
1	Month 1 (Phase 1)	Session 1	Strategic Inattention to Symptoms (shojo-fumon): “Let’s see how it goes”	Patient worked alone; asked permission to continue working individually. Vicious psychic interaction cycle disrupted.
2–8	Months 1–3 (Phase 1)	Sessions 2–8	Maintaining side-by-side stance; observing patient’s behavior without symptom-focused dialogue	Gradual reduction in explicit avoidance behavior; began observing group activities.
9	Month 4 (Phase 2)	Session 9 [Key session]	Role suggestion: proposed patient serve as ‘secretary’ after observing smartphone research behavior	Patient accepted role; attention shifted from internal anxiety to external task. Desire for Life (sei-no-yokubo) elicited.
11	Month 5 (Phase 2)	Session 11 [Key session]	Emotional reframing: guilt about joining group discussions reframed as latent desire to cooperate	Patient accepted reframing; began participating in group discussions more actively.
17	Month 7 (Phase 2)	Session 17	Validation of participation despite physical discomfort (arrived late due to train discomfort)	Reported finding group interaction enjoyable. Purpose-driven Action stance emerging.
—	Months 8–10 (Gap)	No sessions (outpatient follow-up only)	No formal nursing interventions; monthly psychiatric follow-up maintained	Patient A autonomously initiated part-time employment (customer-facing retail, ~3–4 h/shift, 2–3 days/week).
18	Month 11 (Phase 3)	Session 18 [Key session]	Validation of autonomous employment decision	Reported starting part-time job. Increased autonomous decision-making capacity.
21	Month 12 (Phase 3)	Session 21	Validation of experiential insight	“When I’m playing table tennis, I don’t notice the gaze of others.” Attention shifted from self to task.
29	Month 14 (Phase 3)	Session 29 [Final session]	Validation of self-regulatory decision: “It’s important that you judged for yourself to rest.”	Fulfilled day-duty officer role after arriving late. Self-adjustment skills consolidated. Purpose-driven Action stance established.
—	Month 17 (3-month follow-up)	Post-program follow-up	Multidisciplinary monitoring via peer support group	Part-time employment ongoing. Enrolled in peer support group for program graduates.

Clinical timeline of the Morita therapy-based short-care intervention for Patient A. Note: Session numbering and phase boundaries follow the nursing process analysis described in Section 2.4. Month 1 = first session of the short-care program (Year 20XX). The four-month gap (Months 8–10) represents a period of no formal program attendance; monthly outpatient psychiatric follow-up was maintained during this interval.

## Data Availability

The data presented in this study are available on request from the corresponding author. The data are not publicly available due to privacy or ethical restrictions related to the nature of psychiatric case reports.

## Abbreviations

The following abbreviations are used in this manuscript:

DSM-5	Diagnostic and Statistical Manual of Mental Disorders, 5th Edition
SAD	Social Anxiety Disorder
GAF	Global Assessment of Functioning
LSAS	Liebowitz Social Anxiety Scale
HQ-25	Hikikomori Questionnaire-25
ACT	Acceptance and Commitment Therapy
CBT	Cognitive Behavioral Therapy
DBT	Dialectical Behavior Therapy
